# O.4.2-11 Effects of habit formation interventions on physical activity habit strength: meta-analysis and meta-regression

**DOI:** 10.1093/eurpub/ckad133.186

**Published:** 2023-09-11

**Authors:** Haoming Ma, Aoqi Wang, Runyuan Pei, Meihua Piao

**Affiliations:** Chinese Academy of Medical Sciences & Peking Union Medical College, China, People's Republic of; haoming_ma@student.pumc.edu.cn; Chinese Academy of Medical Sciences & Peking Union Medical College, China, People's Republic of; haoming_ma@student.pumc.edu.cn; Chinese Academy of Medical Sciences & Peking Union Medical College, China, People's Republic of; haoming_ma@student.pumc.edu.cn; Chinese Academy of Medical Sciences & Peking Union Medical College, China, People's Republic of; haoming_ma@student.pumc.edu.cn

## Abstract

**Background:**

Interventions aimed at promoting physical activity (PA) behavior through habit formation pathways are gaining popularity, as they differ from conventional interventions that rely on intention pathways. Past research has established a positive correlation between PA habits and behavior. However, the efficacy of current interventions designed to form PA habits and improve PA automaticity is not yet fully ascertained. Additionally, the intervention components that significantly impact the effectiveness of these interventions are yet to be determined.

**Methods:**

We conducted a search of three databases (PubMed, Embase, and Cochrane Library) from January 2000 to December 2022, with a focus on interventions for developing PA habits. Two independent authors conducted paper selection, quality assessment, data extraction, and coding of behavior change techniques (BCTs). The effect size of interventions was calculated using standardized mean difference. Subgroup analyses were carried out based on follow-up duration, delivery method, sample characteristics, and theory. Furthermore, we employed meta-regression to investigate the association between BCTs and PA habits.

**Results:**

Ten eligible studies with relatively high quality were included in the final data set. Characteristics of studies varied in intervention sample and delivery way. The habit formation interventions significantly increased PA habit (SMD=0.31, 95% CI 0.14 - 0.48, P<.001) compared to the control groups. Subgroup analysis demonstrated that the duration of follow-up≤12 weeks have a higher effect size on PA habit than the duration > 12 weeks. Meta-regression revealed that problem solving has a significant positive association with effectiveness improvement (β = 0.36, 95% CI 0.17–0.55), while social reward is linked with a reduction in effectiveness (β = -0.40, 95% CI -0.74–0.06).

**Conclusions:**

Our findings reveal that habit formation interventions are effective in fostering PA habit. Future studies could leverage the insights form this study to optimize the intervention design and achieve better effectiveness.

